# The administration of hydrogen sulphide prior to ischemic reperfusion has neuroprotective effects in an acute stroke model

**DOI:** 10.1371/journal.pone.0187910

**Published:** 2017-11-21

**Authors:** Chul-Woong Woo, Jae-Im Kwon, Kyung-Won Kim, Jeong-Kon Kim, Sang-Beom Jeon, Seung-Chae Jung, Choong-Gon Choi, Sang-Tae Kim, Jinil Kim, Su Jeong Ham, Woo-Hyun Shim, Yu Sub Sung, Hyun Kwon Ha, Yoonseok Choi, Dong-Cheol Woo

**Affiliations:** 1 Asan Institute for Life Sciences, Asan Medical Center, Songpa-gu, Seoul, Republic of Korea; 2 Department of Radiology, Asan Medical Center, Songpa-gu, Seoul, Republic of Korea; 3 Department of Neurology, Asan Medical Center, Songpa-gu, Seoul, Republic of Korea; 4 Medical Research Institute, Gangneung Asan Hospital Gangneung-si, Gangwon-do, Republic of Korea; Henry Ford Health System, UNITED STATES

## Abstract

Emerging evidence has suggested that hydrogen sulfide (H_2_S) may alleviate the cellular damage associated with cerebral ischemia/reperfusion (I/R) injury. In this study, we assessed using ^1^H-magnetic resonance imaging/magnetic resonance spectroscopy (^1^H-MRI/MRS) and histologic analysis whether H_2_S administration prior to reperfusion has neuroprotective effects. We also evaluated for differences in the effects of H_2_S treatment at 2 time points. ^1^H-MRI/MRS data were obtained at baseline, and at 3, 9, and 24 h after ischemia from 4 groups: sham, control (I/R injury), sodium hydrosulfide (NaHS)-30 and NaHS-1 (NaHS delivery at 30 and 1 min before reperfusion, respectively). The total infarct volume and the midline shift at 24 h post-ischemia were lowest in the NaHS-1, followed by the NaHS-30 and control groups. Peri-infarct volume was significantly lower in the NaHS-1 compared to NaHS-30 and control animals. The relative apparent diffusion coefficient (ADC) in the peri-infarct region showed that the NaHS-1 group had significantly lower values compared to the NaHS-30 and control animals and that NaHS-1 rats showed significantly higher relative T2 values in the peri-infarct region compared to the controls. The relative ADC value, relative T2 value, levels of N-acetyl-L-aspartate (NAA), and the NAA, glutamate, and taurine combination score (NGT) in the ischemic core region at 24 h post-ischemia did not differ significantly between the 2 NaHS groups and the control except that the NAA and NGT values were higher in the peri-infarct region of the NaHS-1 animals at 9 h post-ischemia. In the ischemic core and peri-infarct regions, the apoptosis rate was lowest in the NaHS-1 group, followed by the NaHS-30 and control groups. Our results suggest that H_2_S treatment has neuroprotective effects on the peri-infarct region during the evolution of I/R injury. Furthermore, our findings indicate that the administration of H_2_S immediately prior to reperfusion produces the highest neuroprotective effects.

## Introduction

Blood flow reperfusion is considered to be the most important intervention to preserve neurologic function when managing patients with acute cerebral stroke. The infusion of thrombolytics such as recombinant tissue-type plasminogen activator (rt-PA) either intravenously (i.v.) or through endovascular catheterization are standard options to manage reperfusion [1, 2]. However, the reperfusion of ischemic tissue is often associated with tissue damage due to various factors such as inflammatory responses, oxidative stress, and excitotoxicity generated by excessive glutamate release [3].Therefore, many research groups have attempted to develop novel methods for reducing cerebral ischemic/reperfusion (I/R) injury [3–5].

Hydrogen sulfide (H_2_S) has for many decades been primarily considered a pungent toxic gas and an environmental hazard [6]. A number of studies on H_2_S have reported that it has a broad range of physiological and pathophysiological functions, including the regulation of neuronal activity, induction of angiogenesis, and vascular relaxation [7–10]. Over the previous decade, there have also been several reports that H_2_S exerts neuroprotective effects in animal models of cerebral I/R injury, by inhibiting oxidative stress, inflammation, and apoptosis [11–15]. However, only a limited number of studies have involved the administration of H_2_S prior to reperfusion. Furthermore, there have been no reports on the optimal administration time of H_2_S in a cerebral stroke model which is a major point of consideration.

In our current study, we aimed to evaluate the neuroprotective effects of H_2_S when administered prior to reperfusion in a rat cerebral acute stroke model, and also investigate whether the timing of H_2_S treatment influences its neuroprotective effects via a systematic analysis of temporal evolution data generated using vivo ^1^H-MRI/MRS and histology.

## Materials and methods

### Ethics statement

This study was carried out in strict accordance with the guidelines of the National Institutes of Health. All of the procedures were performed following approval by the Institutional Animal Care and Use Committee of the Asan Medical Center (IACUC Number: 2015-14-157). All surgery was performed under isoflurane anesthesia. All the animals were carefully monitored by the trained individuals who can assess the animal pain behavior. Animal euthanasia was planned if the animal showed continuous pain related behavior; however, no animal showed the unrelieved severe pain related behavior during the experimental period.

### Transient middle cerebral artery occlusion (tMCAO) model

We used male Sprague-Dawley rats (eight weeks old, n = 48; weight = 280–310 g; Orient Bio, Pyeongtaek, Republic of Korea). All animals were individually housed in standard plastic cages and maintained on a 12-h light-dark cycle (lights on at 08:00 A.M.) at an ambient temperature of 24.0–25.0°C, with free access to food and water.

The transient middle cerebral ischemic occlusion (tMCAO) model was used to generate I/R injury in rats. Rats were initially anaesthetised with 5% isoflurane and maintained with 2–3% isoflurane during surgery. Middle cerebral artery (MCA) occlusion, using a previously described method of intraluminal vascular occlusion [16], was performed for 60 min to induce ischemia. The MCA occlusion was then relieved to induce reperfusion. In particular, an 18–20-mm 4–0 suture thread (Ethylon surgical monofilament polyamide; Ethicon, Livingston, UK) with a fire-polished tip (diameter, 0.38–0.40 mm) was advanced from the external carotid artery into the lumen of the internal carotid artery until it blocked the origin of the MCA. After 60 min, the inserted intravascular thread was removed gently.

To identify animals wherein the tMCAO model was successfully established, the regional cerebral blood flow (CBF) was monitored before and after MCA occlusion by using a laser Doppler flow (LDF) monitoring device (VMS-LDF, Moor Instruments, Devon, UK). For the placement of the LDF probe, a burr hole (diameter, 2 mm) was drilled 2 mm posterior and 6 mm lateral to the bregma, with care being taken not to injure the underlying dura mater [17]^.^ Rats that did not show a significant CBF reduction after MCA occlusion (at least 70% decrease from the baseline value) were excluded from the experimental group [18]. Sham-operated rats were manipulated in the same way without MCA occlusion.

### Experimental groups

Sodium hydrosulfide (NaHS, Sigma-Aldrich, St. Louis, MO) dissolved in saline (25 μmol/kg of NaHS dissolved in 2.5 ml of saline) was used as an H_2_S donor. Normal saline (2.5 ml) was used for the vehicle. The drug and vehicle were administrated via intravenous injection.

Rats were randomly divided into 4 groups (n = 8 per group): (1) sham-operated group with no I/R modelling or injection of the vehicle (n = 8), (2) control group with I/R modelling and injection of the vehicle (n = 8), (3) NaHS-30 group with I/R modelling and injection of the drug at 30 min before reperfusion (n = 8), and (4) NaHS-1 group with I/R modelling and injection of the drug at 1 min before reperfusion (n = 8). If death or insufficient infarction occurred, additional rats were included to meet the sample size number.

### MRI and ^1^H-MRS

**Fig 1:**
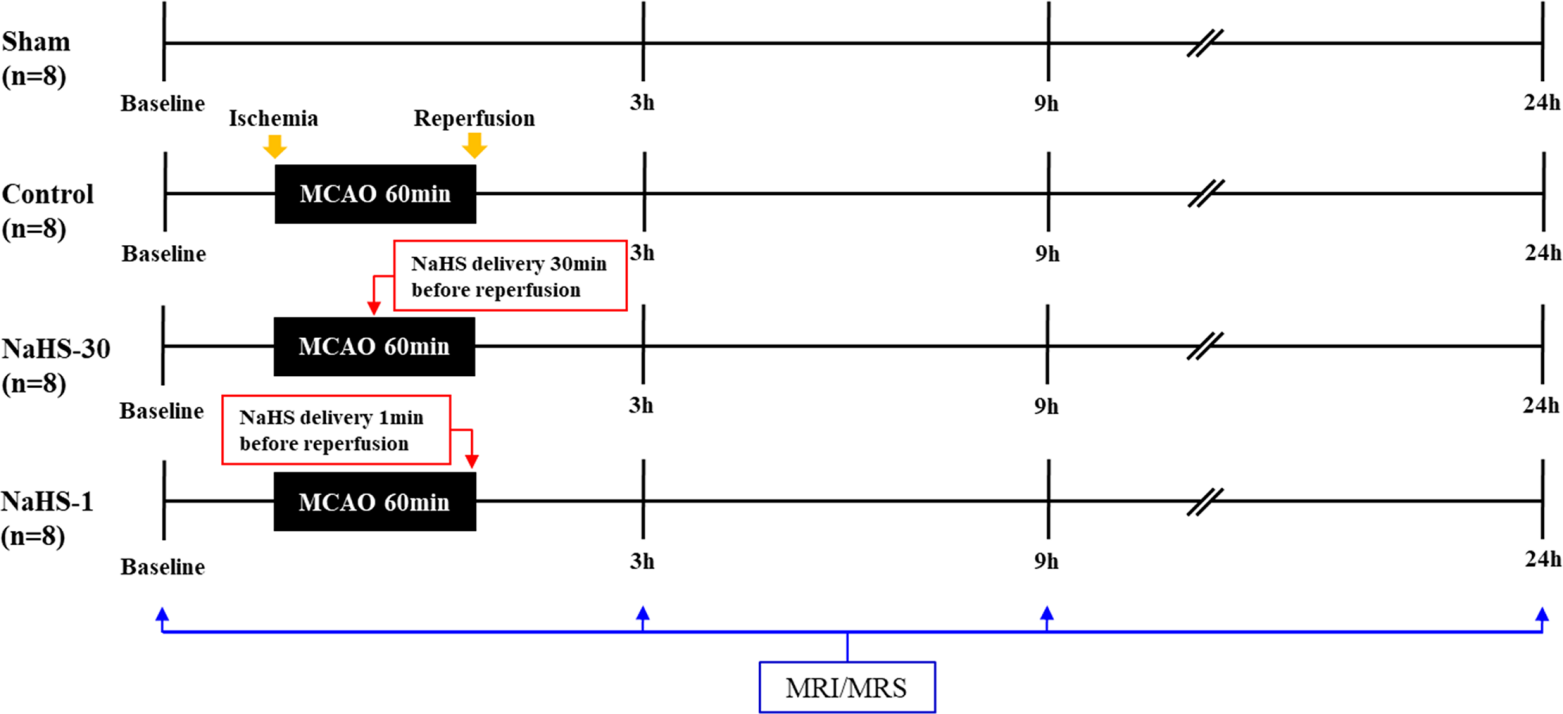
Summary of NaHS administration and MRI/MRS acquisition times in the experimental groups.

^1^H-MRI/MRS was obtained at baseline (i.e. before I/R modelling) and at 3, 9, and 24 h after I/R modelling (Fig 1). MRI and ^1^H-MRS were conducted using a 9.4T/160 mm animal MR system (Agilent Technologies, Santa Clara, CA). A 72-mm birdcage volume coil was used for excitation, and a 4-channel phased array surface coil served as the receiving coil. All the animals were anaesthetised through a mask via the spontaneous inhalation of 2.0–2.5% isoflurane in a 1:2 mixture of O_2_:N_2_O. Respiration was monitored and rats were maintained in a normothermic condition at 37.5 ± 0.5°C using an air heater system.

The MRI protocol included T2-weighted images (T2-WIs), T2 maps, and spin-echo diffusion weighted images (DWIs). T2-WIs were acquired with a fast spin-echo sequence (TR, 4000 ms; k-zero, 3; echo spacing, 10.98 ms; 32 segments; echo train length, 8; effective TE, 32.95 ms; averages, 1; matrix, 256 × 256; field of view, 30 × 30 mm; and slice thickness, 1.0 mm, no gap). T2 map images were acquired using a multi-echo multi-slice (MEMS) sequence with the following parameters: TR, 3000 ms; TE, 10–150 ms; 15 echoes; averages, 1; matrix, 128 × 128; and slice thickness, 1 mm, no gap. Furthermore, the DWI parameters were as follows: TR, 2000 ms; TE, 22.67 ms; averages, 1; matrix, 128 × 128; slice thickness, 1 mm, no gap; and b-values, 0 and 1000 s/mm^2^. Quantitative ADC maps were created on a voxel-wise basis, with a linear least-squares fit on the logarithm of the signal intensity vs. the b-value for each diffusion direction. The geometrical imaging parameters (i.e., number and orientation of slices, FOV) of the T2 maps and DWIs were the same as those used on T2-WIs.

**Fig 2:**
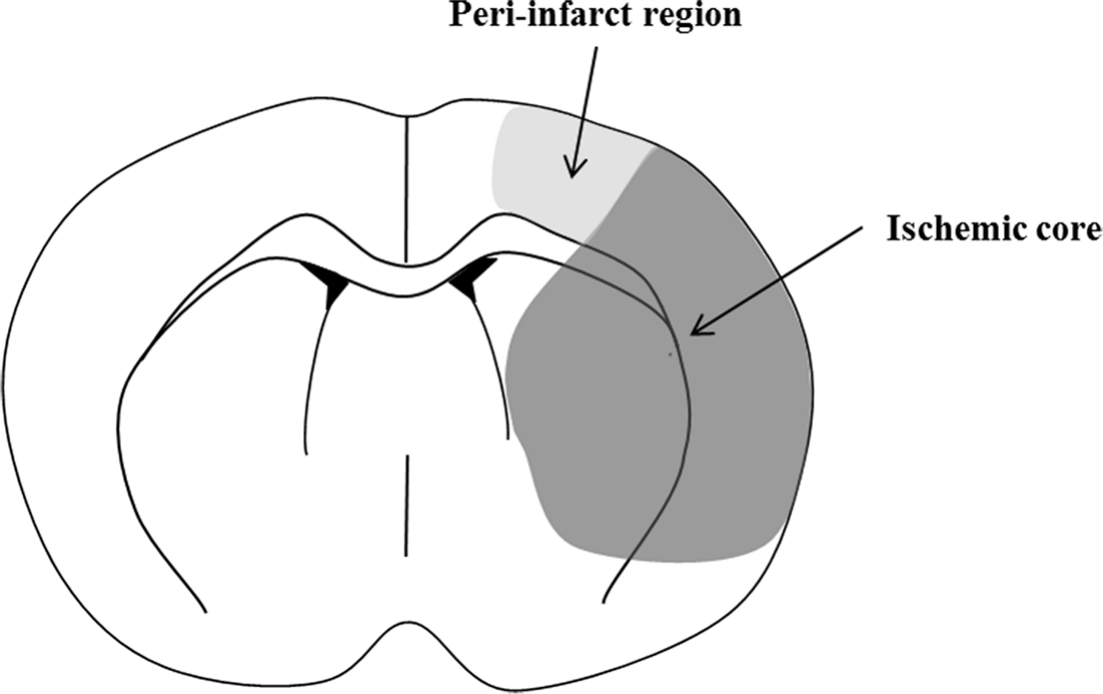
Diagram of the regions of the ischemic core and the peri-infarct region.

^1^H-MRS was performed to detect the metabolites in vivo and monitor the temporal changes caused by the stroke. In particular, the N-acetyl-L-aspartate (NAA) concentration and the combination score of NAA, glutamate (Glu), and taurine (Tau) (NAA + Glu + Tau, NGT) have been proposed as markers of neuronal density and viability in stroke [19, 20]. The MR spectra of standard brain metabolites were collected from a single voxel of 3.5 × 2 × 1.6 mm^3^ in the ischemic core (lateral caudo-putamen and somato-sensory cortex) and peri-infarct region (primary motor cortex and somato-sensory cortex) in the slice, according to the diagram suggested previously by Zhao et al [4] (Fig 2). For single voxel localisation of ^1^H-MRS images, we used point resolved spectroscopy sequence (PRESS) with the variable power RF pulses with optimised relaxation delays (VAPOR) method (TR/TE, 5000/13.47 ms; spectral width, 5 kHz; number of averages, 256; data points, 2048). Respiration gating was used for DWI scan acquisitions, and the total scan time was <90 min.

### MRI analysis

**Fig 3:**
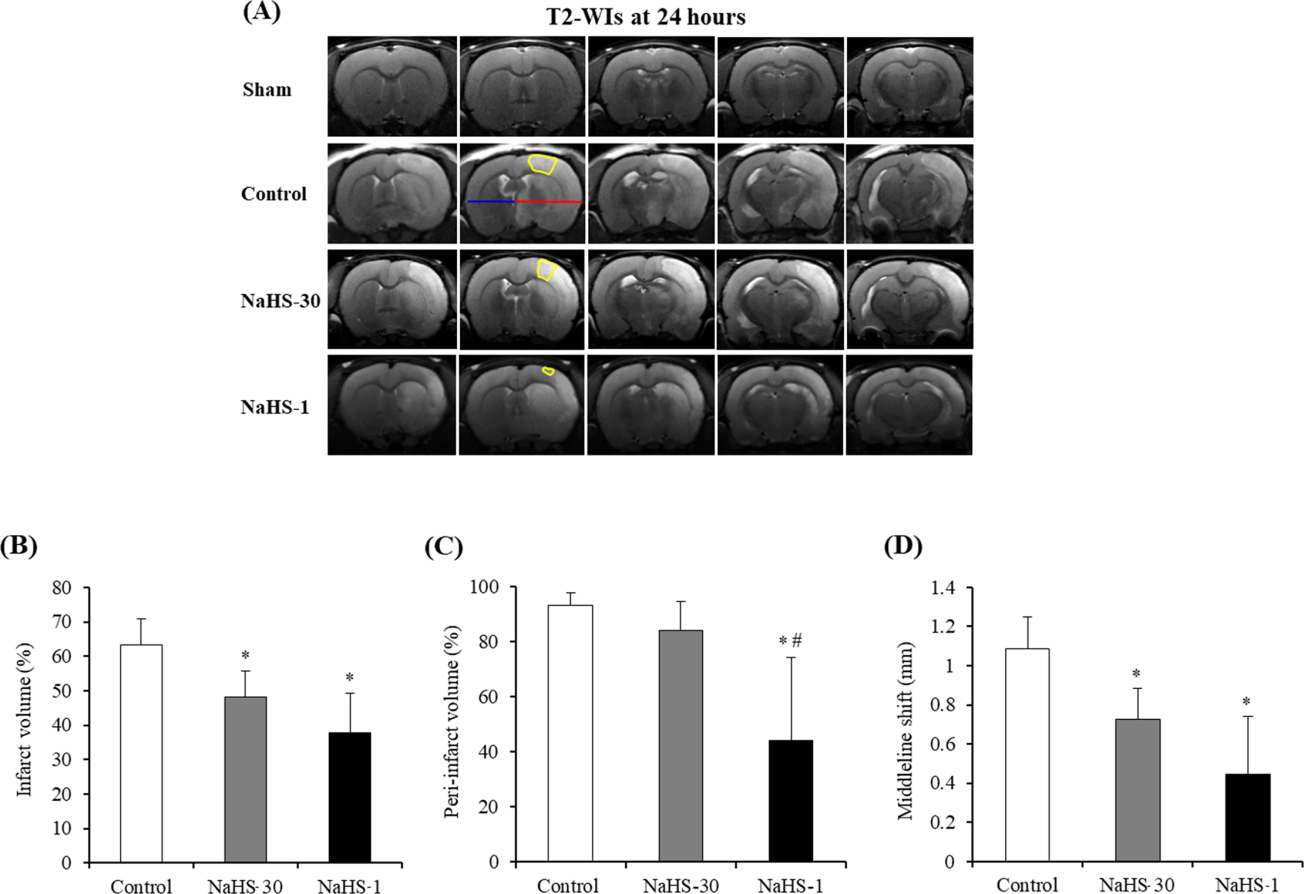
Infarct volumes and midline shift (MLS). (A) The peri-infarct volume was measured from the peri-infarct region (yellow region) on T2-WIs at 24 h after ischemia in representative rats from each group. To calculate the MLS, the distance between the outer border of the cortex and the middle of the third ventricle was measured from the ipsilateral (red line) and contralateral (blue line) sides. (B -D) The total infarct volume (B), peri-infarct volume (C) and MLS (D) were lowest in the NaHS-1 group, followed by the NaHS-30 and control groups. The peri-infarct volume was significantly lower in the NaHS-1 group than in the NaHS-30 group. Data are presented as mean ± standard deviation (n = 8 rats in each group). *P < 0.001 vs. the control group, ^#^ P < 0.001 vs. the NaHS-30 group.

All MRI data were assessed by an observer blinded to the grouping information. MRI analysis was conducted using ImageJ software (National Institutes of Health, Bethesda, Maryland; http://rsbweb.nih.gov/ij/). The total infarct volume at 24 h after ischemia was measured on T2-WI scans using the 2D volumetry technique, which involves the summation of the infarct volume measured from each slice [21]. The peri-infarct volume at 24 h after ischemia was also measured on T2-WI scans, which involves the infarct volume measured from the peri-infarct region in the slice (Fig 2).The midline shift (MLS) quantification method was used to determine the space-occupying effect of the cerebral edema [22]. This method was performed on T2-WI scans at 24 h after ischemia, where the position of the third ventricle could be determined clearly in all animals. The distance between the outer border of the cortex and the middle of the third ventricle was measured from the ipsilateral (A) and contralateral (B) sides (Fig 3). Measurements were obtained at the level of the maximum lateral displacement of the ventricle. MLS was calculated using the following equation: MLS = (A − B)/2, as described previously [23].

**Fig 4:**
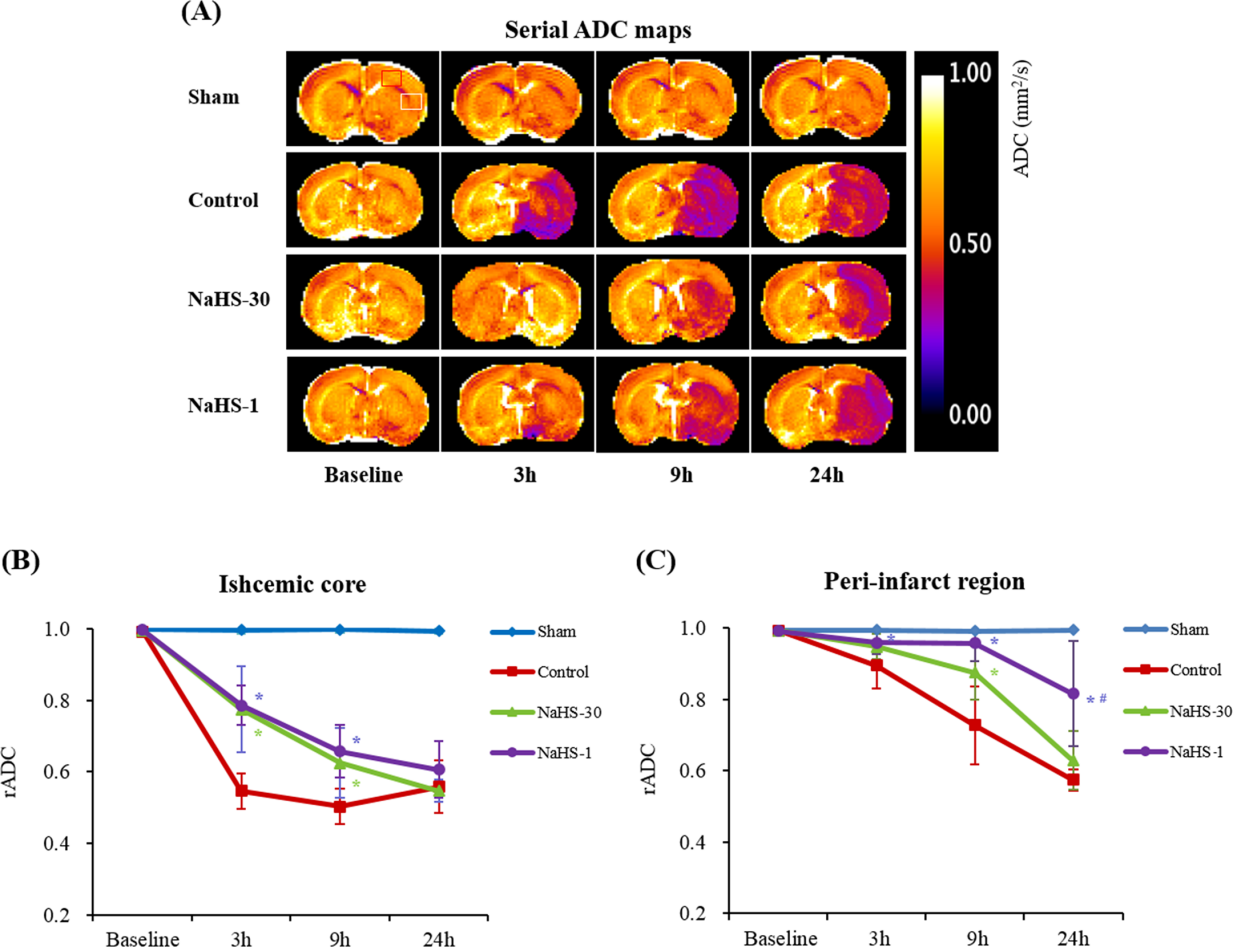
Effect of NaHS treatment on the ADC value. (A) The mean ADC values were measured in the ipsilateral (ischemic core [white square] and peri-infarct [red square]) and contralateral regions on the ADC maps at baseline and at 3, 9, and 24 h after ischemia. (B, C) The rADC of the ischemic (B) core and (C) peri-infarct regions were plotted against time. Data are presented as a mean ± standard deviation (n = 8 rats in each group). * P < 0.001 vs. the control group, ^#^ P < 0.001 vs. the NaHS-30 group.

**Fig 5:**
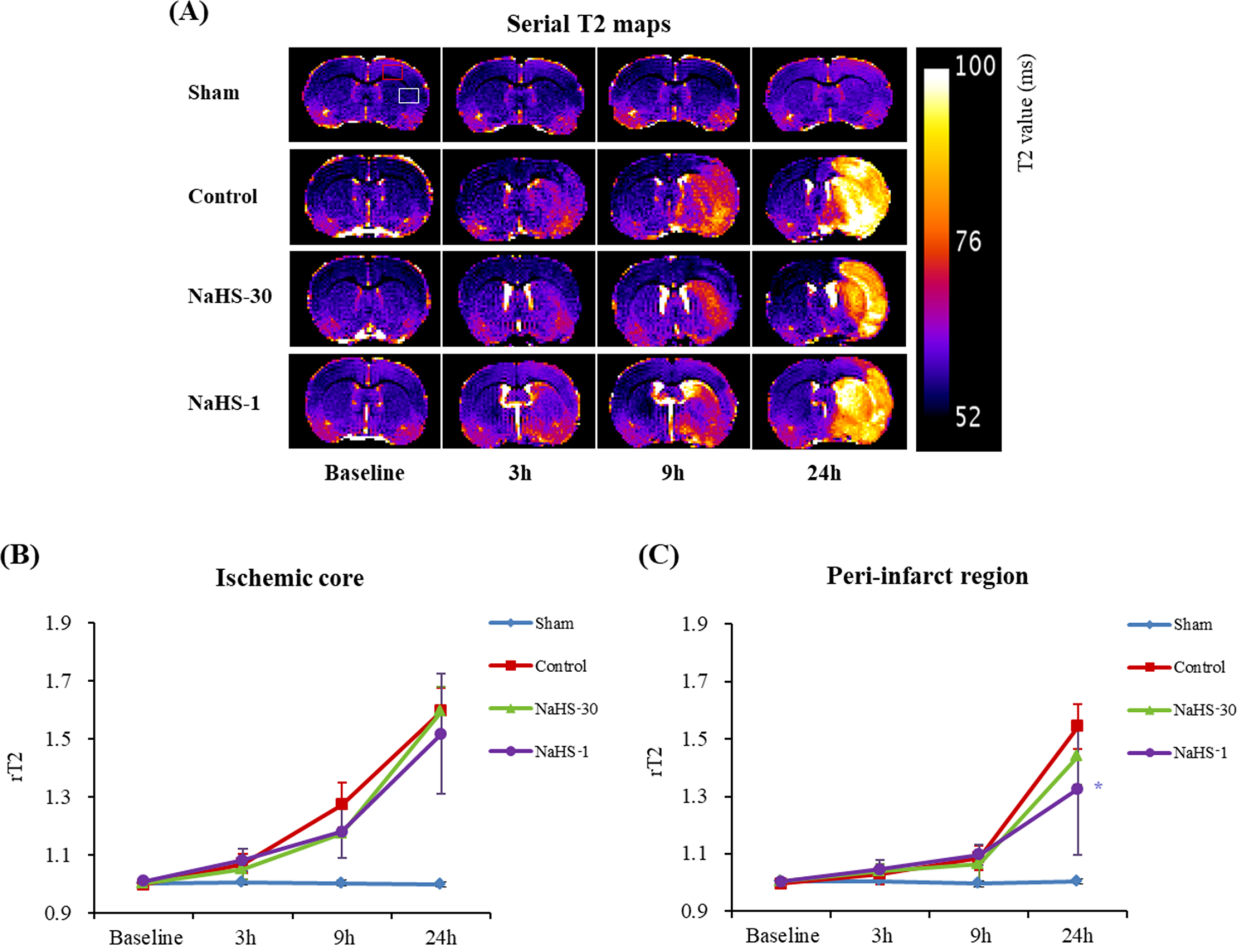
Effect of NaHS treatment on the T2 value. (A) The mean T2 values were measured in the ipsilateral (ischemic core [white square] and peri-infarct [red square]) and contralateral regions on serial T2 maps at baseline and at 3, 9, and 24 h after ischemia. (B, C) The rT2 of the ischemic (B) core and (C) peri-infarct regions were plotted against time. Data are presented as mean ± standard deviation (n = 8 rats in each group). * P < 0.001 vs. the control group, ^#^ P < 0.001 vs. the NaHS-30 group.

The degree of ischemic injury was evaluated by measuring the ADC and T2 values on a respective map. The regions-of-interest (ROIs) were located in the ischemic core and peri-infarct region of the ipsilateral hemisphere, and also in the corresponding ROIs in the contralateral hemisphere (Figs 4 and 5). Thereafter, the relative ADC (rADC) and T2 (rT2) values were calculated as ratios i.e., ipsilateral value/contralateral value, as previously decribed [24].

### ^1^H-MRS analysis

The resulting spectra were processed as described by Lei et al [25]. Briefly, absolute quantification was obtained using a linear combination analysis method (LC Model ver.6.0, Los Angeles, CA). The MR spectra were considered acceptable if the signal-to-noise ratio (SNR) was ≥8 and the standard deviation (Cramér-Rao lower bounds, CRLB) of the spectral fit for the metabolite was <30%. The concentrations of NAA (cNAA) and NGT (cNGT) were measured in the ischemic core and peri-infarct regions at baseline and at 3, 9, and 24 h after ischemia.

### Terminal transferase d-UTP nick-end labelling (TUNEL) assays

The extent of apoptosis in the damaged tissues was assessed using the TUNEL assay. For this purpose, we used the ApopTag Peroxidase In Situ Apoptosis Detection Kit (Chemicon, CA).

The rats were sacrificed and fixed by cardiac perfusion with 4% paraformaldehyde at 24 h after I/R modelling. The brain tissue was isolated and fixed in 4% paraformaldehyde for 3 days. The fixed brain tissues were then sectioned coronally (thickness, 3 μm) and mounted on prechilled glass slides coated with poly-L-Iysine. Tissue sections were incubated in a dry oven for 1 h at 60°C. The tissues were treated with a working solution containing reaction buffer and enzyme (7:3) mixture for 1 h at 37°C, followed by an anti-digoxigenine peroxidase conjugate for 30 min at room temperature. The tissues were then treated with the DAB substrate (1:50) for 5 min in a dark room, followed by haematoxylin for 2 minutes in the dark room for cell staining.

The TUNEL-stained sections were examined under a microscope (ZEISS, HAL100, 200× magnification) and photographed. A brown stain in the nucleus represents an apoptotic cell. The number of TUNEL-positive cells and total cells were counted using ImageJ software in 3 randomly chosen fields from the ischemic core and peri-infarct regions. The percentage of TUNEL-positive cells relative to the total cell count was used to evaluate the apoptosis rate.

### Statistical analysis

All data are expressed as means ± standard deviation. Statistical analysis was performed using SPSS version 13.0 software (SPSS, Chicago, IL). The CBF reduction, total infarct volume, MLS, rADC, rT2, cNAA, cNGT, and apoptosis rate in multiple groups were compared using one-way analysis of variance with multiple post-hoc comparison with Scheffe's method. Differences with a P value <0.05 were considered statistically significant.

## Results

### tMCAO modelling

The success rate of tMCAO modelling was 60% (24 included animals, with 40 receiving surgery). The causes for exclusion were as follows: death during operation [control (n = 2) and NaHS-1 (n = 2) groups], death after operation [control (n = 4), NaHS-1 (n = 1), and NaHS-30 (n = 3) groups], and no infarction or insufficient infarction generated [NaHS-1 (n = 2) and NaHS-30 (n = 2) groups]. The 4 rats without any infarction were excluded after laser Doppler monitoring indicated insufficient CBF reduction, or after MRI performed at 3, 9, and 24 h after I/R modelling did not show any signs of infarction. All the included rats in the NaHS-1, NaHS-30, and control groups exhibited marked decreases in the regional CBF after ischemia i.e., > 70% reduction, compared to the baseline regional CBF (Table 1). The rats in the sham-operated group did not show any reduction in the CBF. There was no significant difference in CBF reduction among the NaHS-1, NaHS-30, and control groups (81% ± 5%, 81% ± 5%, and 79% ± 5%, respectively, P = 0.9019, one-way ANOVA).

**Table 1:** rCBF changes before and after the onset of ischemia.

Group	rCBF before ischemia (perfusion unit)	rCBF after ischemia (perfusion unit)	rCBF reduction (%)	p-value
Control	168 ± 18	35 ± 11	79 ± 05	
NaHS-30	177 ± 02	34 ± 10	81 ± 05	0.9019 ^a^
NaHS-1	179 ± 12	35 ± 09	81 ± 05	0.9019 ^a^

^a^ *P* = 0.9019 vs. the control group.

### Infarct volumes and MLS on T2-WI scans

Fig 3 shows the infarct lesions that were evident on T2-WI scans at 24 hours after I/R modelling in the representative rats from each group. The total infarct volume, peri-infarct volume, and MLS at 24 h after I/R modelling were lowest in the NaHS-1 group, followed by the NaHS-30 and control groups, which is indicative of the neuroprotective effect of NaHS. In the total infarct volume and MLS analyses, one-way ANOVA post-hoc tests showed that both NaHS treatment groups had significantly lower values than the control group (NaHS-30 v.s. Control, P < 0.001; NaHS-1 v.s. Control, P < 0.001), however, no significant difference was observed between the NaHS-1 and NaHS-30 groups. In the peri-infarct volume analysis, the results showed that the NaHS-1 treated group had significantly lower values than either the control or NaHS-30 treated groups (NaHS-30 vs. NaHS-1, P < 0.001; NaHS-1 vs. Control, P < 0.001).

### ADC and T2 values

Figs 4 and 5 show the evolution of the lesions from ADC maps and T2 maps at baseline, and at 3, 9, and 24 h after I/R modelling in the representative rats from each group. The ischemic/infarcted area in the ipsilateral hemisphere showed low ADC values and high T2 values, compared to the contralateral hemisphere, thus reflecting the degree of cytotoxic/vasogenic cerebral oedema. In the sham-operated group, the ADC and T2 values did not differ over time. On serial ADC maps, the rADC of the ischemic core and peri-infarct region decreased over time in the control, NaHS-1, and NaHS-30 groups. On serial T2 maps, the rT2 of the ischemic core and peri-infarct region increased over time in the control, NaHS-1, and NaHS-30 groups. The rADC and rT2 of the ischemic core reached a similar level at 24 h after I/R modelling, with no significant difference found among the control, NaHS-1, and NaHS-30 groups (P > 0.05, one-way ANOVA). In contrast, the rADC and rT2 values from the peri-infarct region differed significantly between the groups at 24 h after I/R modelling (P < 0.001, one-way ANOVA). Post-hoc tests revealed the highest value in the NaHS-1 group, followed by the NaHS-30 group and control group, with significant differences between the groups (adjusted P < 0.05 for each comparison, Scheffe's test). These results suggest that the strongest neuroprotective effects, particularly those for the preservation of the peri-infarct region (i.e., penumbra), were in the NaHS-1 group.

### cNAA and cNGT on ^1^H-MRS

**Fig 6:**
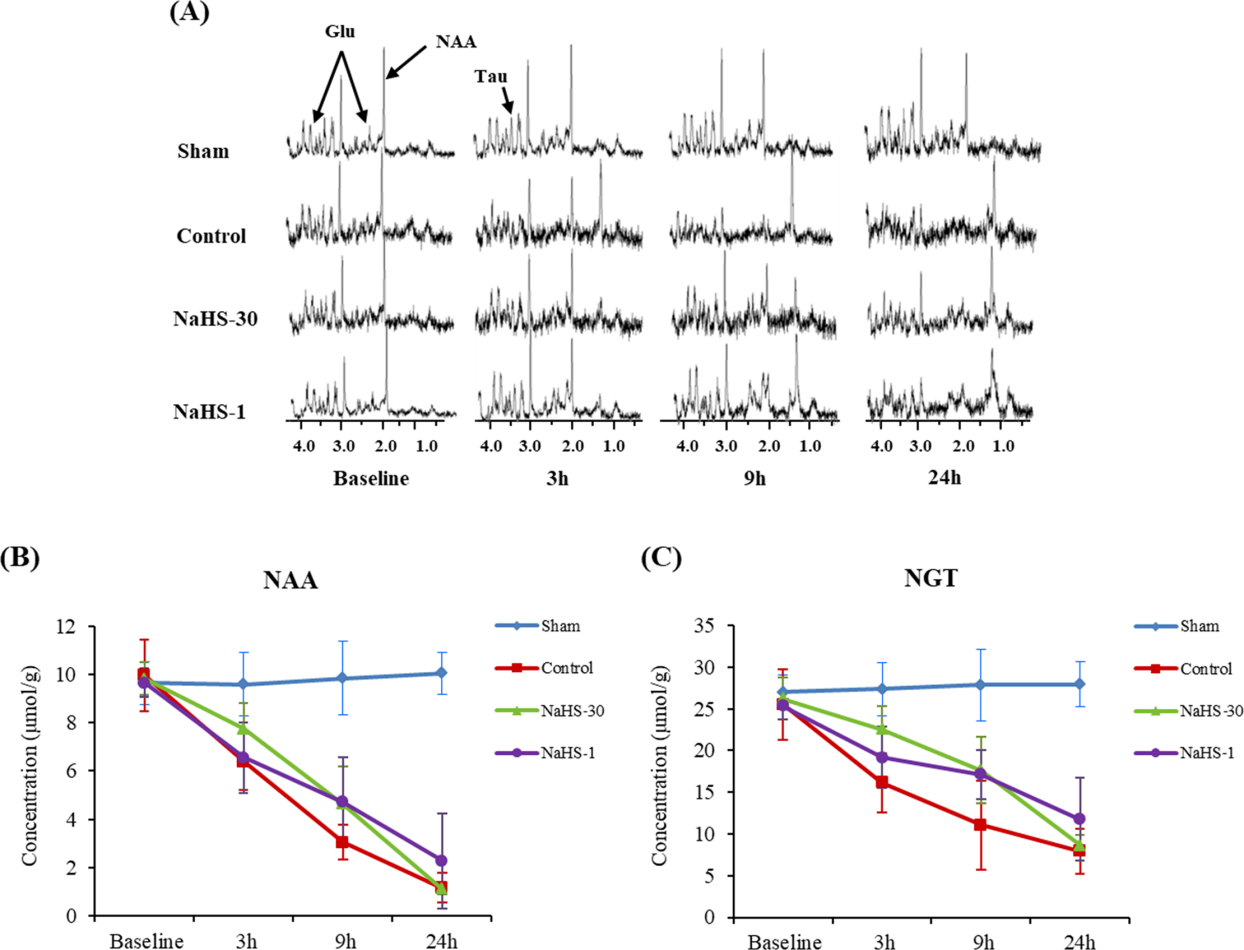
cNAA and cNGT changes in the ischemic core. (A) MR spectra at baseline and at 3, 9, and 24 h after ischemia. (B, C) The cNAA (B) and cNGT (C) values were plotted against time. The cNAA and cNGT levels decreased over time in the control, NaHS-1, and NaHS-30 groups and reached a similar level at 24 h after I/R modelling in all three groups. The cNAA and cNGT values did not differ significantly between the three groups at any time point.Data are presented as mean ± standard deviation (n = 8 rats in each group).

**Fig 7:**
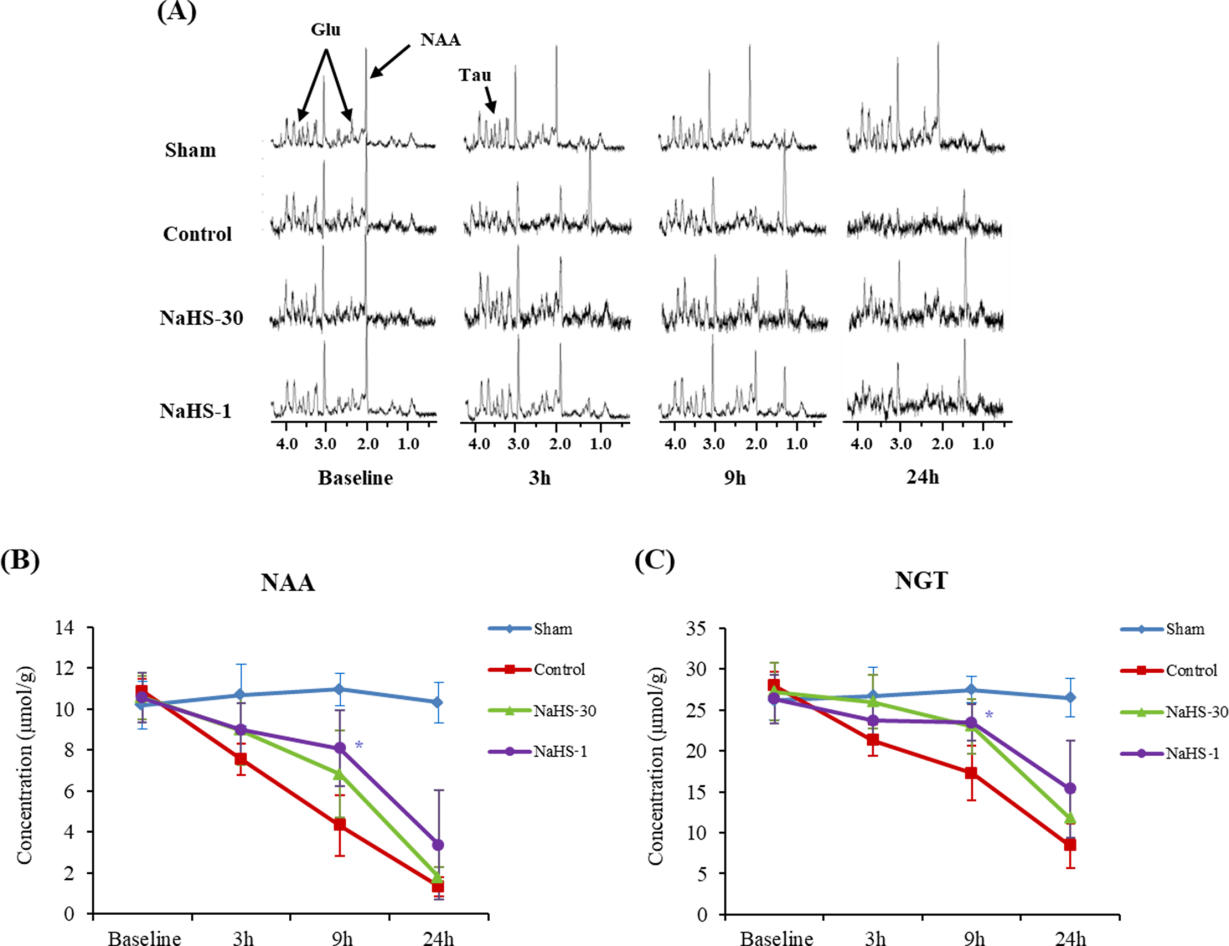
cNAA and cNGT changes in the peri-infarct region. (A) MR spectra at baseline and at 3, 9, and 24 h after ischemia. (B, C) The cNAA (B) and cNGT (C) values were plotted against time. The cNAA and cNGT levels decreased over time in the control, NaHS-1, and NaHS-30 groups and reached a similar level at 24 h after I/R modelling in all three groups. The cNAA and cNGT values did not differ significantly between the three groups at any time point other than 9 h after I/R modelling. Data are presented as mean ± standard deviation (n = 8 rats in each group). * P < 0.001 vs. the control group.

Figs 6 and 7 show the MR spectra for the ischemic core and peri-infarct region, respectively, at baseline and at 3, 9, and 24 h after ischemia in representative animals from each group. On serial ^1^H-MRS analysis of the ischemic core and peri-infarct regions, the cNAA and cNGT levels were found to decrease over time in the control, NaHS-1, and NaHS-30 groups, reflecting the temporal evolution of the metabolites associated with I/R injury. In the ischemic core and peri-infarct regions, the cNAA and cNGT values did not differ significantly between the groups at the 3 h and 24 h time points (one-way ANOVA, P > 0.05), although post-hoc tests indicated that the peri-infarct region of the NaHS-1 animals had significantly higher cNAA and cNGT values than those of the control and NaHS-30 groups at 9 h.

### Extent of apoptosis

**Fig 8:**
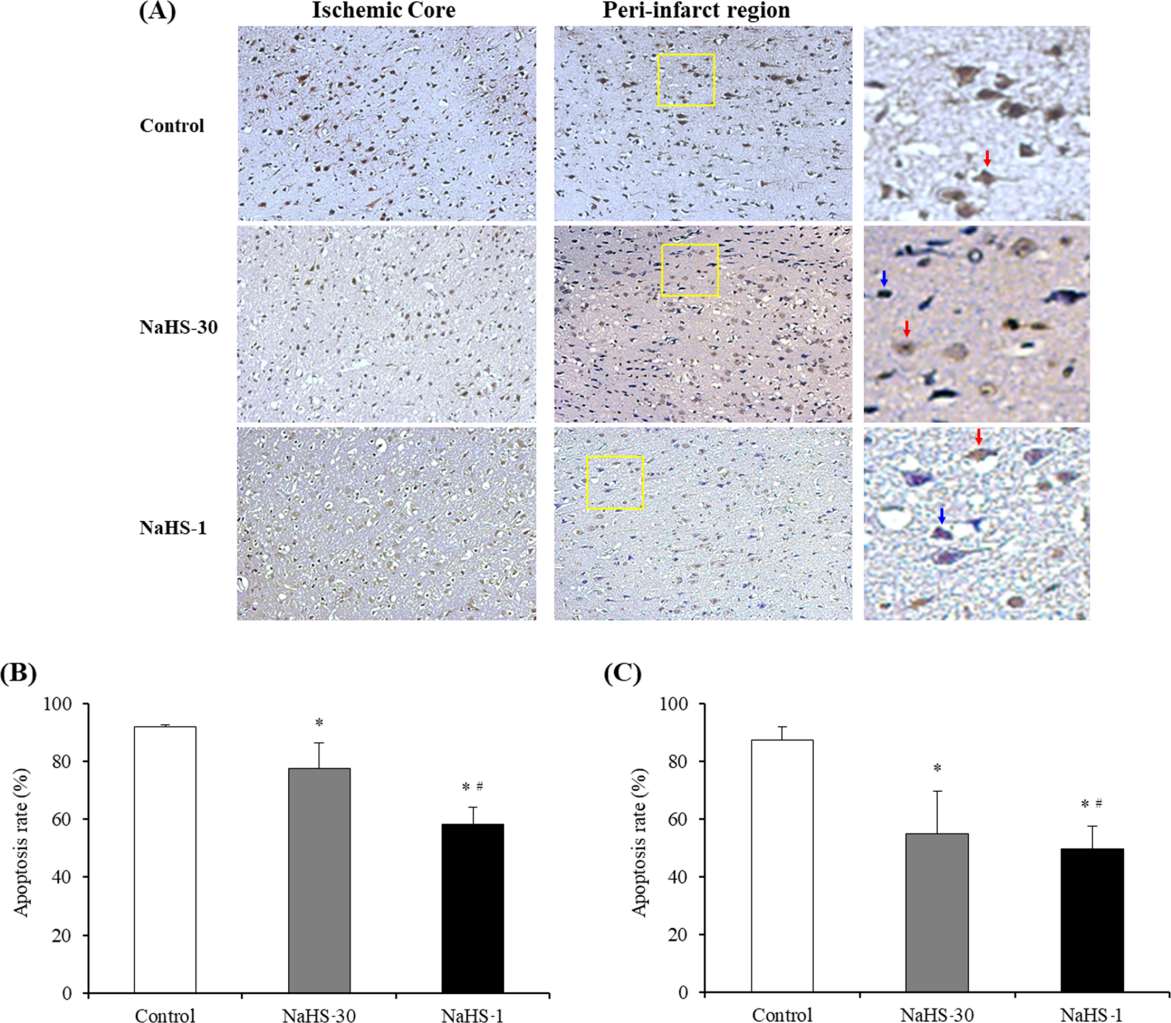
NaHS treatment suppresses apoptosis. (A) Representative photomicrographs of TUNEL staining results in the ischemic core and peri-infarct regions (magnification 200×). Red arrow indicates apoptotic cells; blue arrow indicates viable cells. (B, C) Quantification of the effect of NaHS treatment on the apoptosis rate in the ischemic (B) core and (C) peri-infarct regions. Data are presented as mean ± standard deviation (n = 8 rats in each group). * P < 0.001 vs. the control group, ^#^ P < 0.001 vs. the NaHS 30 group.

Positively stained apoptotic cells (round with brown nuclei) were analyzed in the NaHS-1, NaHS-30, and control groups (Fig 8). In the ischemic core and peri-infarct regions, the lowest apoptosis rate was observed in the NaHS-1 group, followed by the NaHS-30 and control groups, with these differences showing significance (P < 0.001, one-way ANOVA). Post-hoc tests further revealed that the apoptosis rate was significantly lower in the NaHS treatment groups than in the control group (P < 0.001). Moreover, a significant difference in the ischemic core and peri-infarct region apoptosis rates was observed between the NaHS-1 and NaHS-30 groups (P < 0.001).

## Discussion

Consistent with the results of prior studies [11, 14, 21, 26], we found in our current analysis that H_2_S has therapeutic effects against I/R injury in the brain. Using T2-WI analyses, which is the best sequence for anatomic evaluations (total infarct volume, peri-infarct volume and MLS), H_2_S treated rats showed reduced infarct volumes and MLS values compared with control animals at 24 h post-ischemia. Moreover, our NaHS-1 treatment group showed significantly better neuroprotective effects compared with the NaHS-30 and control groups. Although H_2_S treated groups showed overall therapeutic effects compared to the control group, our data showed some discrepancies and conflicts in therapeutic efficacies according to the analysis methods and the measurement times. We assumed that it arose from the heterogeneities of pathological progression for each model entity; however, if the refined analysis technology in the stroke research is introduced rather than the current analysis method, we believe that this problem can be amended.

Using rADC and rT2 measures that respectively represent the formation of cellular edema [27] and vasogenic edema [28], we found from our current analysis that H_2_S administered at 1 min before reperfusion showed a significant decrease in these values at the peri-infarct sites which are widely regarded as salvagable (penumbra). With regard to the influence of H_2_S administration timing, our results indicated that applying this treatment at 1 min before reperfusion produced better neuroprotective effects in the peri-infarct region compared with a far earlier administration (30 min) Consistently, some previous studies that have used a myocardial infarction model have reported that H_2_S treatment at the onset of reperfusion led to a significant decrease in the myocardial infarct size [29, 30]. However, no previous study has assessed the effect of H_2_S administration at 1 min before reperfusion in a cerebral I/R model.

We postulated that the pharmacokinetic properties of H_2_S may underlie these timing effects. When reperfusion occurs, the blood that is newly returning into the ischemic area carries the initial abundant inflammatory response and oxidative stress factors. In addition, previous reports indicate that H_2_S reaches its highest level within a few minutes after the administration [31]. It thus seems reasonable that exposure to H_2_S immediately before reperfusion would have more protective effects against initial reperfusion damage. It must be noted however that we only examined a single dose of H_2_S in our analysis (25 μmol/kg, NaHS) that had been suggested previously by Li et al. and Ren et al [21, 32]. Moreover, no study to date has assessed the relationship between the blood concentration and the anti-oxidant effects of H_2_S. Further studies are therefore needed to more precisely evaluate the relationship between the timing of H_2_S delivery and its subsequent blood concentration.

^1^H-MRS can sensitively detect metabolites in vivo and monitor their temporal changes during stroke. NAA, which is predominantly present in neurons, has been proposed as a marker of neuronal density and viability [19]. NGT is used as a predictive marker of ischemic severity [20]. In our current results, the NAA and the NGT levels in the peri-infarct region of the subject rats were higher in the NaHS-1 group than in the controls at 9 h post-ischemia. However, there were no differences in these parameters in any of the animal groups at 24 h post-ischemia. Possible reasons for this finding may be the evolution of stroke injury and the technical limitations of the analytical methods we used. As oxidative stress and glutamate-mediated excitotoxicity are sustained during the evolution of cytotoxic/vasogenic edema [33–35], disruption of the blood brain barrier (BBB) and the plasma membrane ion transport function gradually occurs. Therefore, although the protective effects of H_2_S can attenuate oxidative stress and glutamate-mediated excitotoxicity, it appears that this will only delay the eventual cell death that arises from continuous damage to different areas of the brain. Notably, we only measured the NAA and the NGT levels in limited regions of the rat brain and more wide ranging analysis on the neuroprotective mechanism of H_2_S will therefore be needed. An investigation using multi-voxel MRS may further elucidate this phenomenon.

## Conclusions

We have evaluated the protective effects of H_2_S administration prior to reperfusion and assessed the relationship between the timing of H_2_S delivery and the subsequent neuroprotective effects against I/R injury in a rat model. Neuroprotective effects of H_2_S against edema and apoptosis were observed. Furthermore, our results support our hypothesis that the therapeutic timing of H_2_S exposure affects its neuroprotective impact against I/R-induced cerebral injury. Nevertheless, further studies on the precise time-dependence of H_2_S efficacy against cerebral I/R injury and the mechanism underlying its neuroprotective effects are needed to assess its potential clinical application.

## Supporting information

S1 Table Result values of infarct volume, peri-infarct volume, and MLS in Fig 3. (XLSX)

S2 Table Result values of rADC in Fig 4. (XLSX)

S3 Table Result values of rT2 in Fig 5. (XLSX)

S4 Table Result values of cNAA and cNGT (ischemic core) in Fig 6. (XLSX)

S5 Table Result values of cNAA and cNGT (peri-infarct region) in Fig 7. (XLSX)

S6 Table Result values of apoptosis rate in Fig 8. (XLSX)

S1 File NC3Rs ARRIVE guidelines checklist. (PDF)
